# Accuracy of Microimplant Placement Using a 3D Guide Plate for Orthodontic Anchorage

**DOI:** 10.1155/2023/9060046

**Published:** 2023-06-26

**Authors:** Fangyong Zhu, Lian Ji, Chen Zhou, Yannan Cao, Zhifei Chen, Xiangbing Wu, Jianming Zou, Yufeng Gao

**Affiliations:** ^1^Department of Stomatology, Affiliated Hospital of Jiangnan University, 1000 Hefeng Road, 214000 Wuxi, China; ^2^Department of Stomatology, Wuxi People's Hospital, 214000 Wuxi, China

## Abstract

This study aimed to design a three-dimensional (3D) guide plate using computer-aided design and a 3D printing system for precise implantation of microimplants for orthodontic treatment and investigate the accuracy and feasibility of a 3D guide plate in clinical practice. A total of 30 microimplants were placed in 15 patients in the Department of Stomatology, Affiliated Hospital of Jiangnan University. Before surgery, DICOM data from cone-beam computed tomography (CBCT) scans and STereoLithography data from the 3D model scan were imported to 3Shape Dental System. Data fitting and matching were performed, and 3D guide plates were designed primarily focusing on the thickness of guide plates, amount of concave compensation, and dimensions of the ring. Assist implantation method was used to place the microimplants, and postoperative CBCT images were used to evaluate the position and implantation angle. The feasibility of placing microimplants and precise implantation guided by the 3D guide plate. CBCT data before and after the placement of microimplants were compared. Regarding the secure positioning of microimplants based on CBCT data, 26 implants were categorized as Grade i, four as Grade ii, and none as Grade iii. No loosening of microimplants 1 and 3 months after surgery was reported. The implantation of microimplants is more accurate under the guidance of a 3D guide plate. This technology can achieve accurate implant positioning, thus ensuring safety, stability, and improved success rates after implantation.

## 1. Introduction

The implantation angle of microimplants has always been a focus of clinical research. Several studies have shown that microimplants placed in an oblique direction can increase the bony contact and insertion torque, produce better stability and retention, and significantly improve the biological and biomechanical stability of microimplants [1–3]. Although the depth of oblique implantation is lesser than that of vertical implantation, the length across the cortical bone is increased [4]. In clinical practice, the implantation angle is usually determined based on the clinician's experience. Therefore, a method to objectively determine the implantation angle and position is necessary [5–7]. The three-dimensional (3D) guide plate is using computer-aided design. The cone-beam computed tomography (CBCT) scan data in DICOM format and the oral scan data in STereoLithography (STL) format were imported into 3Shape Implant Studio software. The 3-point alignment with 3Shape Implant Studio software was used to align the angles or cusps of three teeth of the same name in pairs from CBCT and intraoral scan images, such as 11 incisor angles, 13 cusps, and 25 buccal tips of tooth. CBCT is the basis for fitting intraoral scan. Inaccurate data acquisition may lead to deviations in the final position of microimplants. The intraoral soft tissue data that could not be accurately obtained by CBCT and hard tissue data such as root data that could not be obtained by intraoral scan, which can be both presented on the same 3D model. In the existing 3D position, the position of the anchorage nail was set. In this position, the guide ring of the anchorage nail guide plate is set. The position and orientation of the microimplant are the key factors to determine the stability of the microimplant. At present, the clinical manipulation is mainly free hand, and there is a lot of randomness. Some scholars have tried to use CBCT to make a 3D positioning guide plate by rapid prototyping technology to determine the insertion point and angle of microimplant screws. This practice has improved the success rate of microimplant screws. However, in clinical practice, microimplant screws are often implanted after bracket bonding. The designed full-coverage digital microimplant guide plate often cannot be fully positioned because the teeth are bonded with brackets, which affects the accuracy [8, 9].

Our technology has three advantages: one is that this method can apply the offensive surgical method to the microimplant anchorage implantation under the guidance of digital 3D guide plate, and evaluate the accuracy of the application of the offensive 3D guide plate. Another is that this method can simplify and formulate the standard process of microimplant anchorage implantation directed by 3D guide plate, and also formulate the producing plan in detail. The third advantage is that the DICOM files related to the CBCT and STL files of the model are imported into the 3Shape Dental System software, and the relevant data fitting and matching operations are completed to design the 3D template. At the same time, the position and angle of microimplant anchorage are evaluated by postoperative CT scan.

Hence, we aimed to design a computer-aided 3D guide plate using CBCT scans and patient intraoral scan data after fitting for the placement of microimplants and investigate the feasibility and accuracy of this technology in clinical practice [10–12].

## 2. Patients and Methods

### 2.1. Patients

From November 2020 to November 2021, 15 patients requiring bilateral buccal microimplant anchorage for orthodontic treatment in the upper and lower posterior edentulous areas in the Department of Stomatology, Affiliated Hospital of Jiangnan University, were enrolled. There were six males and nine females, with an average age of 17.11 ± 6.28 years. The study design was approved by the ethics committee of The Affiliated Hospital of Jiangnan University.

### 2.2. Inclusion Criteria

Patients with healthy periodontal tissues, no history of orthodontic treatment or permanent tooth extraction (except third molar extraction), no history of congenital tooth loss, and those who provided informed consent and met the relevant requirements of the medical ethics committee.

### 2.3. Exclusion Criteria

Patients with diabetes mellitus, poor physique, infectious diseases, severe wear and tear of teeth, active periodontitis, craniomaxillofacial deformities, jaw space-occupying lesions, and a history of systemic diseases affecting bone metabolism. CBCT scans of the oral cavity of all patients were taken before surgery, and data were collected in DICOM format. CBCT data must meet the following requirements to be included in the analysis: slice thickness of 0.5 mm, reconstruction layer spacing of 0.5 mm, scanning domain of 140–170 mm, and a frame tilt angle of 0°. CBCT is the basis for fitting intraoral scan. Inaccurate data acquisition may lead to deviations in the final position of microimplants. In the CBCT taken by the patient, the anchorage nail is located between the two roots of the teeth, and the distance between the proximal and distal is between 0.5 and 1 mm. Therefore, it is required that CBCT should not jitter or glose due to occlusion and other problems during the shooting. At the same time, if the CBCT is blurred, it will directly affect the three-position fitting of the CBCT and the patient's intraoral scan data. The error value of data integration directly determines the accuracy of implant implantation. The field of view of CBCT was 8 × 8 mm, and nonmodular CT was used. The time of CBCT was consistent with the time of taking the mold. CBCT data were exported as third-party volumetric multifile DCM format data (the number of files varies according to the thickness of CBCT, but a minimum of 200 files or more should be required).

A 3D scan was performed on the preoperative impression model derived from the patient (Hangzhou Preclinical scanner), and the STL data of the model scan were obtained. 3shape design software cannot be imported if the following conditions are not met. The position of the microimplant was selected in the adjacent gingival tissue according to the need, and connected by the adjacent teeth across. (Note: The direction of the undercut of the tooth that fills the working side is downward from the occlusal plane, trying to be evenly divided on the buccolingual side of the tooth.) The frame selection range was 2-3 anterior and posterior teeth to be implanted, and the connecting rod across was usually 3-4 mm. Finally, the STL file of the implant guide plate was generated.

### 2.4. 3D Reconstruction and Design

DICOM data from CBCT scans and STL data from the model scan were imported to 3Shape Dental System to fit and match the images. Important anatomical structures of the mandible, such as the shape of the mandibular neural tube and the position of the mental foramen, were determined. Based on the condition of the alveolar bone and nerve distribution, the microimplant implantation scheme was designed.

### 2.5. Guide Plate Design

PROTECT V microimplants (diameter, 1.6 mm; length, 8 mm) from Zhejiang Pute Company were used in the study. 3Shape Dental System was used to design the 3D guide plate after the superposition of the images. The safe distance between the implant apex and tooth root was greater than 0.5 mm, and the distance between the implant neck and alveolar bone was certain. The implant position and angle were designed to be in the range of 60°–90° with the long axis of the tooth, parallel to the near and far middle tooth root, without damaging the adjacent anatomical structures such as the maxillary sinus, and was located halfway between the roots of the near and far middle teeth. Microimplants are usually supported by two or three adjacent teeth. Therefore, the design of the adjacent tooth support was adopted. In cases of a bracket and archwire design, it was avoided. To ensure an accurate implantation angle, a ring was designed on the 3D guide plate where the height and diameter of the ring were determined based on the height and diameter of the microimplant. The ring was designed as follows: distance from the bottom of the microimplant to the top of the ring = length of the microimplant plus gingival thickness plus compensation distance from the guide ring to the gingiva plus guide ring distance. These parameters ensured that the angle and position were always the same during the implantation of multiple microimplants.

### 2.6. 3D Guide Printing

**Figure 1:**
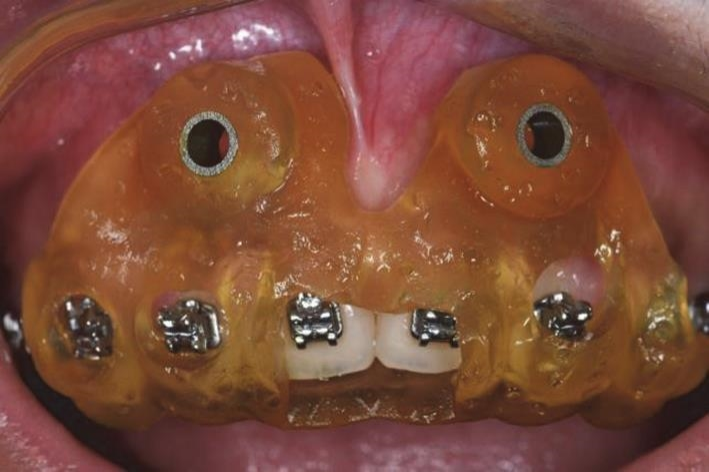
3D surgical guide plate for anterior teeth.

A 3D printer (FORMLABS 2) was used to print the guide plate. After the 3D guide was generated, it was soaked in 95% ethyl alcohol for 5 min. After rinsing it with normal saline, visual light was used for 5 min to solidify the 3D plate (Figure 1).

The guide plate was tried on the patient a few days before surgery to ensure its fit and stability. The observation hole confirmed that the tooth crown and gingiva were completely in line with the tissue surface of the guide plate without warping or swinging. Resin protrusions on the guide plate were adjusted, and the guide plate was removed and disinfected for later use.

### 2.7. Surgical Technique

**Figure 2:**
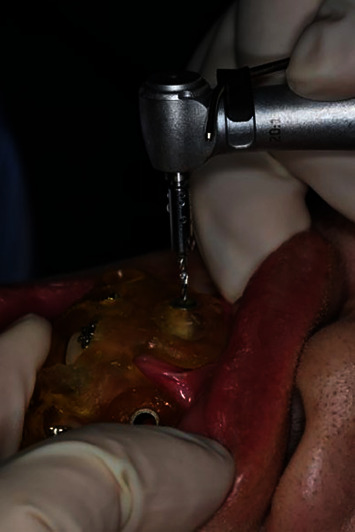
Implantation process of the micoimplant in the anterior maxillary area.

**Figure 3:**
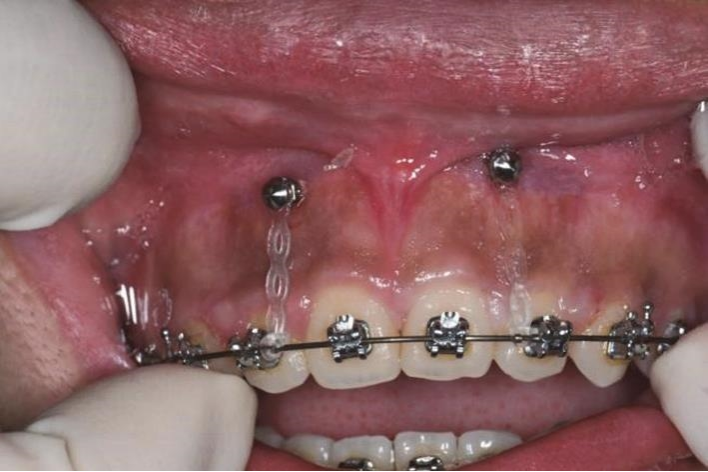
Immediately after loading the anterior microimplant.

**Figure 4:**
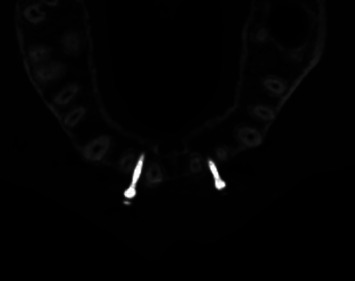
Computed tomography scan of the anterior maxillary area immediately after implantation.

The surgical area was disinfected with 1% iodine tincture and was locally anesthetized using attevacaine. The patient was draped, and the 3D guide was placed in the oral cavity. Microimplants were placed using the assist implantation technique (Figure 2; Table 1), which was performed as follows: the self-developed drill was used to prepare the osteotomy site according to the order of implantation. First, a mucous membrane trephine was used to puncture the gingiva, and the pioneer drill was selected according to the length of the implant and was used to drill into the cortical bone. Next, the microimplant was implanted into the prepared osteotomy site. During the procedure, the cortical bone was slowly drilled with a needle, and saline irrigation was used to cool the osteotomy site to protect the surrounding tissue (Figures 3 and 4).

## 3. Evaluation

### 3.1. Accuracy of Microimplant Implantation

**Figure 5:**
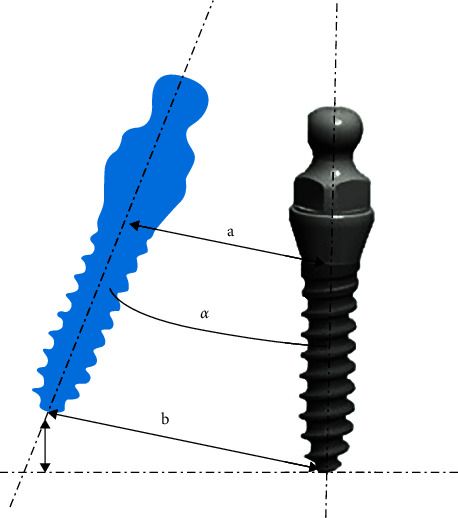
Accuracy of implantation angle and microimplant position using the 3D guide plate.

All patients underwent CBCT scanning immediately after surgery. All data were saved in DICOM format. Preoperative and postoperative CBCT images were superimposed and matched. In Figure 5, the blue-colored implant represents the virtual implant location, the gray-colored implant represents the actual implant location, a represents the neck deviation between the actual and virtual implants, b represents the root deviation between the actual and virtual implants, and *α* represents the angular deviation between the actual and virtual implants, measured in the mesiodistal and buccolingual directions [13].

### 3.2. The Deviation between Preoperative Virtual Implant and Actual Implant at the Neck, Apical Area, and Implantation Angle

### 3.3. Evaluation of Safety after Implantation

After the implantation of microimplants, information from CBCT scans was used to grade the security of the position between the microimplants and adjacent roots of the two teeth, which was divided into three grades. Grade i denoted that the microimplant was located between the two adjacent roots, approximately equidistant from each root; Grade ii denoted that the microimplant located between the two adjacent roots was deflected to one side, although the implant was not in contact with the tooth root. Grade ⅲ denoted that the microimplant was in contact with the adjacent tooth root. The total sample size was 30. Each clinician observed each sample, and three clinicians were required to independently observe each sample [14].

### 3.4. Evaluation of Microimplant Stability after Implantation

A force was immediately applied after implantation. Microimplants on the left and right sides of the maxillary arch were examined 1 and 3 months after surgery, and the loosening of the microimplants was recorded.

### 3.5. Statistical Analysis

The experimental data are expressed as mean ± standard deviation, and SPSS version 18.0 statistical analysis software was used to analyze the data. To evaluate the reliability of measurements, data of 30 microimplants were tested twice, one day apart, and the paired *t* test was performed on the measured values. A *P*-value of >0.05 was not considered statistically significant.

## 4. Results

### 4.1. Accuracy Analysis

All implant sites were ideal and similar, owing to the use of a 3D guide plate. The average deviation at the neck of the microimplant postoperatively and that of the original preoperative design was 0.13 ± 0.4 mm, respectively (*P* > 0.05), while the average deviation between the tooth root and the original design was 0.21 ± 0.60 mm (*P* > 0.05), which was not statistically significant. The mean deviation of the implantation angle was 1.15° ± 3.21° and 1.25° ± 3.71° in the mesiodistal and buccolingual directions, respectively (*P* > 0.05), showing no statistical significance (Table 1). These findings indicate that the position of the microimplant postoperatively is consistent with that of the preoperative design (Table 2).

### 4.2. Safety and Stability of Microimplants after Implantation

CBCT images taken immediately after implantation showed that the safety rating of 26 microimplants was Grade ⅰ, four was Grade ⅱ, and none was Grade ⅲ.

## 5. Discussion

Regarding the computer-aided design of guide plates was reported in some previous works [15–17]. For example, Park et al. [18] reported that to minimize root contacts, microimplants need to be inclined distally about 10–20 mm and placed 0.5–2.7 mm distally to the contact pointtom in imizeroot contact according to sites and levels, except into palatal interradicular bone between the maxillary first and second molars. Our study only planned the position of the microimplant implantation according to the clinical experience, while our article not only accurately planned the position of the microimplant implantation through the design and clinical application of the surgical guide plate but also accurately transferred the positioning to the oral cavity, and the completion of clinical precisely operation. In addition, our team just focused the surgical guide plate to locate the supernumerary teeth in last year [19]. But in current study, we specify the diameter and height of the guide ring with the assist method, for the placement of the microimplant. In particular, tapping is also performed when to facilitate the insertion of microimplants. Besides, the function of the guide plate is more refined and convenient for the clinical placement of microimplants in different parts.

Currently, microimplants have been widely used in orthodontic treatment for anchorage. Several studies have started to consider the accuracy of microimplant placement. The greater the role of the microimplant through the attachment of the head. The stability of microimplants is closely related to the quality of the cortical bone. The stress generated during and after implantation is primarily borne by the cortical bone. Marquezan et al. [20] found that cancellous bone plays an important role in the stability of microimplants in the absence of cortical bone. Chen et al. [21] reported that the success rate of microimplant placement in high-angle patients with a large mandibular plane angle was significantly lower than that in equal-angle patients with normal mandibular plane angle or low-angle patients with small mandibular plane angle. Bone density plays a decisive role in the displacement of microimplants [22, 23]. Oblique implantation is a commonly used technique for microimplant placement, but the harder the bone, the more difficult it is to place the implant. A microimplant 3D guide plate, rather than the clinician's experience, can be used to objectively determine the position and angulation of multiple implants to be placed in the maxilla and/or mandible before surgery, which is of practical significance.

Furthermore, attached gingiva is the primary factor that determines the stability of microimplants postoperatively. In this study, the position of the attached gingiva was referenced in the guide plate design by drawing and gently carving a joint line on the 3D model. Previous studies showed that this method could effectively indicate the attached gingiva on the guide plate during implant placement and had no effect on the use of 3D guide plates. Several studies [24] emphasized that microimplants need to be implanted in the attached gingiva to reduce the occurrence of peri-implant inflammation. When the implantation site is not in the attached gingiva, peri-implant inflammation occurs, which ultimately results in the failure of the microimplant.

Root spacing is also one of the key factors in microimplant anchorage. Studies have shown that, after implantation, the failure rate is significantly increased when the implant is too close to the root. Therefore, it is necessary to determine the safe distance between microimplants and the tooth root before implantation. Schnelle et al. [25] suggested that the distance between the dental roots at the implant site should be greater than 3.0 mm. Microimplant placement can be divided into two types: attack and nonattack. The aid type can help control the implantation angle and can be used in multiple areas of the oral cavity [26, 27]. When designing the 3D guide, the BMD around the microimplant is considered. If the patient's BMD is too high, the aid type of implant is used with saline irrigation to decrease the temperature generated during implantation, thus reducing complications. The attack type of microimplant is inserted with some angulation, increasing the contact area with the cortical bone, whereas the self-attacking type can only be placed vertically to avoid slipping along the bone surface.

## 6. Conclusion

Computer-aided design and the 3D printing system were used to generate a 3D guide plate for the placement of microimplants, which are used as anchorage for orthodontic tooth movement. The proposed method was easy to follow, and the position and angulation of microimplants could be accurately calibrated, which can increase the contact area of the microimplant with the cortical bone. The soft tissue on the surface is attached to the gingiva and will not touch the root of the adjacent teeth. Furthermore, a 3D guide plate enables the placement of multiple implants in a single sitting. A ring in the 3D guide plate with fixed inner and outer diameters ensures the accuracy of implant placement. Therefore, by using data from CBCT scans, a 3D guide plate can be generated that helps in determining the position and angulation of microimplants during surgery, which can significantly improve the success rate of microimplants.

## Figures and Tables

**Table 1:** Characteristics of the microimplants used in the study.

Brand	Puter pure titanium microimplant
Composition	Titanium alloy (TC4)
Material shape	Top “a” word groove structure, with a small and round head. The neck is enlarged for attachment suspension and has a cylindrical thread design.
Specifications	Diameter: three types; 1.4 mm (for anterior tooth area), 1.6 mm (between the molar–premolar area on the palatal side), and 2.0 mm (mandibular external oblique line area). Length: 10 mm.
Preparation method	CNC cutting
Physical and chemical properties	Composition: Nickel ≤0.10, without cadmium, beryllium, and lead. The surface of the microimplant is anodized.
Function of the microimplant	For orthodontic anchorage, the microimplant is implanted into maxillary or mandibular bone as a fixed component to resist the forces generated by tooth movement and assist orthodontic tooth movement.
Adverse reactions	Peri-implant diseases, including peri-implant mucositis and peri-implantitis.
The inner diameter of the guide plate sleeve ring	The inner diameter of the guide plate sleeve ring is 4.5 mm, the outer diameter is 5.3 mm, and the length is 5 mm. The upper and lower position of the cuff can be adjusted according to the height of the gingiva.
Guide plate thickness	2.5 mm
Concave compensation	0.75 mm

**Table 2:** Average deviation between the neck, root, angle in near and far, and angle in the buccolingual direction and the original design single sample *t* test, statistically significant difference.

	The average deviation from the original design (mm/°)	*t*	*P*-value
Neck	(0.13 ± 0.40)	1.780	0.085
Root	(0.21 ± 0.60)	1.917	0.065
Angle in near and far	(1.15 ± 3.21)°	1.962	0.059
Angle in buccolingual direction	(1.25 ± 3.71)°	1.845	0.075

## Data Availability

The data used to support this study is available from the corresponding author upon request.

## Abbreviations

CBCT: Cone-beam computed tomography
3D: Three dimensional
SLT: STereoLithography.
